# Mercy Hospital, Chicago

**Published:** 1885-07

**Authors:** 


### CLINICAL REPORTS.

---

MERCY HOSPITAL CLINIC. *Service of* EDMUND ANDREWS, M. D., LL. D. *Professor of Clinical Surgery in Chicago Medical College.*

CASE I. *Total obstruction of the Colon for four months. Colotomy.*—This remarkable case was previously under the care of Dr. Le Duc, of Lagrange, Ills., who sent it here. The patient states that some years ago, while living in England, she had a large tumor removed by way of the vagina while under the influence of chloroform. On awaking she saw the tumor, which appeared to her as large as a "bullock's heart." She had a vague idea that it was perhaps an ovarian tumor, but as their are no marks of incision on the abdomen, it was probably a uterine growth. After the operation there was a gradual diminution of frequency of the fecal evacuations, until they at last occurred only once in a week or two. Some months ago there was an attack of inflammation in the region of the sigmoid flexure. Last winter the movements of the bowels become still less frequent, until in March, 1885, there was only one evacuation, and that was the last she ever had. She continued to take food moderately, retaining it for a time, but always vomiting up the remnants of it at last. Dr. LeDuc witnessed the vomiting repeatedly and pronounced it decidedly stercoraceous, even to having a cylindrical form, as if molded in an intestine. He is of the opinion that the watch kept on her case has been so long and thorough, as to exclude the possibility of a successful hysterical deception. Granting, then, that the history is not erroneous, this woman has been four months without an operation of the bowels. She presented at clinic an appearance of mild invalidism, but by no means of desperate emaciation and exhaustion, and she was able to walk about freely. On examination, the rectum was found absolutely clean of all fecal matter, and Dr. Le Duc had repeatedly examined with the same result. There was no stricture within nine inches of the anus. There was no induration nor tumor to be felt in the abdomen, and there was a moderate layer of fat under the abdominal integument. In consideration of the history, I judged that there was obstruction of the colon at or near the sigmoid flexure. I therefore performed lumbar colotomy, in the usual way, on the left side. I was disappointed to find the colon, when opened, absolutely destitute of any fecal contents. The patient was placed in bed, and experienced a considerable diminution of the tendency to vomit, but the constipation continued. Being in bed, it was easy to prove that there was no hysterical deception this time. However, on the sixth day after the operation, she had three free evacuations from the artificial anus. Another period of inaction ensued, but on the fourteenth day the bowels moved again, and on the fifteenth day they moved once or twice more. The vomiting is slight, but has not altogether ceased. The case is still under observation, and may be the subject of future report.

CASE 2. *Carcinoma of the Pylorus. Laporotomy.*—This patient has a small, hard, and movable tumor between the xiphoid cartilage and the umbilicus. He had been rapidly losing flesh, but still walked about. An exploratory incision was made, with antiseptic precautions, in the linea alba. The tumor lay behind the lesser omentum, compelling me to pass through that membrane. Its base firmly embraced the pylorus, and adhered strongly to four inches of the lower border of the stomach. Exploration with the fingers, showed enlarged and indurated mesenteric glands in the vicinity. As the infection of the glands rendered a cure impossible, it was useless to put the patient to the perils of an excision of the pylorus, so that it was necessary to desist, and close up the incision. The patient recovered from the operation without any signs of peritonitis.

In Vienna the famous excisions of the pylorus performed for this disease are being abandoned. In fact, it is impossible to justify their performance. At present, Billroth is experimenting on a temporary alleviation by opening the small intestine, and stitching it to an orifice in the stomach, thus providing a route for the food, but leaving the disease untouched. Thus far most of the patients appear with great punctuality in the dead house, though a few survive a little longer, until the disease kills them.

CASE 3. *Litholapaxy.*—This patient is a young man in fine health. The stone was very small, measuring less than a centimeter in diameter. On being seized and crushed with a lithotrite, almost every particle of it was drawn out in the jaws of the instrument, so that the evacuator next inserted in due course of the operation washed away only a few fine grains of sand. The patient recovered in a few days. This is my twenty-sixth case of litholapaxy, with only one death.

CASE 4. *Litholapaxy.*—The patient came to the clinic from a distant State, and in an exhausted condition from malarious poisoning complicated with cystitis. He had been mostly confined to his bed for a month and a half. Examination showed a stone in the bladder three-quarters of an inch in diameter, which was crushed and washed out at one sitting under the influence of ether. The operation was done in the presence of warm carbolized water, which by its benumbing influence on the nerves of the bladder, powerfully prevents shock, as well as septicism. Slight inflammation followed with a maximum temperature on the second day of $101\frac{1}{2}°$ F. This rapidly subsided under the use of quinine, morphine and carbolized injections. One of the subsequent examinations detected a small remaining fragment of stone, which being removed, the patient was freed from all trouble, and was able to travel at about the thirty-fifth day.

